# TGF-β-associated extracellular matrix genes link cancer-associated fibroblasts to immune evasion and immunotherapy failure

**DOI:** 10.1038/s41467-018-06654-8

**Published:** 2018-11-08

**Authors:** Ankur Chakravarthy, Lubaba Khan, Nathan Peter Bensler, Pinaki Bose, Daniel D. De Carvalho

**Affiliations:** 1 Princess Margaret Cancer Centre, University Health Network, Toronto, M5G 1L7 ON Canada; 2 Department of Medical Biophysics, University of Toronto, Toronto, M5G 1L7 ON Canada; 3 Ohlson Research Initiative, Arnie Charbonneau Cancer Institute, Cumming School of Medicine, University of Calgary, Calgary, T2N 4N1 AB Canada; 4 Department of Biochemistry and Molecular Biology, Cumming School of Medicine, University of Calgary, Calgary, T2N 4N1 AB Canada; 5 Department of Oncology, Cumming School of Medicine, University of Calgary, Calgary, T2N 4N1 AB Canada; 6 Department of Surgery, Cumming School of Medicine, University of Calgary, Calgary, T2N 4N1 AB Canada

## Abstract

The extracellular matrix (ECM) is a key determinant of cancer progression and prognosis. Here we report findings from one of the largest pan-cancer analyses of ECM gene dysregulation in cancer. We define a distinct set of ECM genes upregulated in cancer (C-ECM) and linked to worse prognosis. We found that the C-ECM transcriptional programme dysregulation is correlated with the activation of TGF-β signalling in cancer-associated fibroblasts and is linked to immunosuppression in otherwise immunologically active tumours. Cancers that activate this programme carry distinct genomic profiles, such as *BRAF*, *SMAD4* and *TP53* mutations and *MYC* amplification. Finally, we show that this signature is a predictor of the failure of PD-1 blockade and outperforms previously-proposed biomarkers. Thus, our findings identify a distinct transcriptional pattern of ECM genes in operation across cancers that may be potentially targeted, pending preclinical validation, using TGF-β blockade to enhance responses to immune-checkpoint blockade.

## Abstract (web summary)

Changes in ECM are of predictive value in pancreatic and colorectal cancer prognosis. Here, the authors perform a pan-cancer analysis and find a subset of ECM genes that is linked to TGF-β signalling signature and is correlated with immunotherapy failure.

## Introduction

The ability to disseminate, invade and successfully colonise other tissues is a critical hallmark of cancer that involves remodelling of the extracellular matrix (ECM) laid down by fibroblasts^1^. Moreover, cancer-associated fibroblasts (CAFs) produce key growth factors and cytokines as components of the ECM that fuel tumour growth, metastasis and chemoresistance and immune response^2–4^. Further, ECM changes also predict prognosis in pancreatic^5^ and colorectal cancers^6,7^.

Here we examine the pan-cancer landscape of ECM gene dysregulation and find that a subset of ECM genes is dysregulated specifically in cancer and is enriched among transcriptional changes that distinguish normal from malignant tissue. We further show that the high expression of this subset of genes is adversely prognostic in pan-cancer analyses. Then, using deconvolution and analyses of transcriptional profiles from dissociated tumour fractions, we show that these genes are modulated in CAFs.

Subsequently, based on multiplatform analysis of The Cancer Genome Atlas (TCGA) data, we correlated these profiles to transforming growth factor (TGF)-β signalling in the tumour microenvironment and show that this transcriptional programme is enriched in immunologically active cancers, suggesting a possible role in immune evasion/adaptation. Finally, we demonstrate that this transcriptional programme predicts responses to immune checkpoint blockade better than mutation burden^8^, cytolytic activity (CYT)^9^, TGF-β expression alone, a CAF signature^10^ or a T cell-inflamed signature^11^. We have thus identified a novel signature of immune evasion that is a potential target for pharmacological modulation and may facilitate effective patient stratification in precision immunotherapy, pending preclinical validation.

## Results

### Definition of a pan-cancer ECM dysregulation profile

Initially, to study ECM gene dysregulation across cancers, we defined a transcriptional signature to distinguish malignant (*n* = 8043) and normal tissues (*n* = 704) accounting for tumour type (*n* = 15) from TCGA and tested for enrichment of an ECM-associated gene-set we curated based on gene ontology terms (Supplementary Table 1, Supplementary Figure 1A). We were motivated to define such a pan-cancer signature given the wide variability of ECM gene transcription per se across tissue types (Supplementary Figure 1B).

**Fig. 1:**
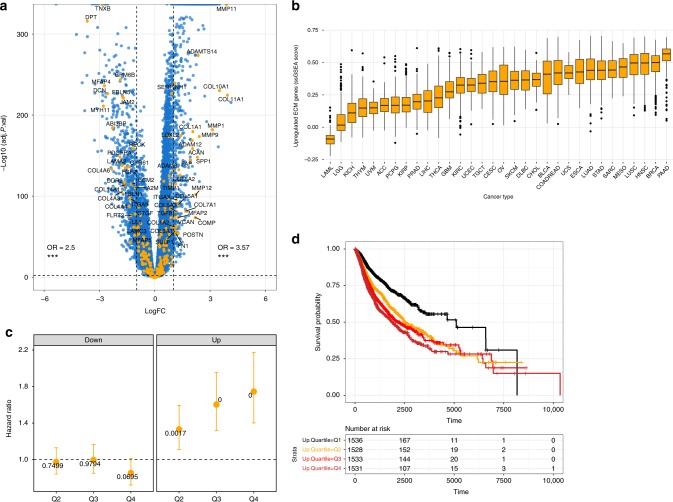
ECM genes are significantly associated with tumorigenesis and prognosis. **a** Volcano plot showing fold changes for genes differentially expressed between cancer and normal samples. ECM genes are enriched in upregulated and downregulated genes. **b** Boxplots of C-ECM-up enrichment scores show variation across tumour types (*n* = 9716, see S1F for C-ECM-down genes). **c** Plot of Cox model coefficients by quartile for C-ECM-up and -down scores pan-cancer, error bars indicate 95% confidence intervals (*n* = 6128). **d** Unadjusted Kaplan–Meier curves showing survival by C-ECM-up-quartile., asterisks indicate statistical significance. ***p < 0.001. On the volcano plot, *y* axis = −log_10_ fold change, *x* axis = test statistic/fold change/Spearman’s Rho. On volcano plots, all enrichment statistics are from Fisher’s Exact Tests

This identified 58 out of the 249 ECM genes represented in the RNA-seq data set to be cancer associated (from hereon referred to as cancer-associated ECM genes (C-ECM genes)) (Supplementary Table 2), comprising 30 out of the 522 upregulated genes, and 28 out of the 644 downregulated genes, representing significant enrichment among both upregulated (odds ratio (OR) = 3.51, *p* < 3.9e−8, Fisher’s Exact Test, two-sided) and downregulated (OR = 2.57, *p* = 3e−5, Fisher’s Exact Test) genes in malignant tissues (Fig. 1a). These C-ECM genes showed generally high correlations and clustering the Pearson correlation matrix of intergene correlations across TCGA cancers segregated them into generally distinct blocks of upregulated and downregulated genes (Supplementary Figure 1C, two sided), suggesting co-regulation.

Notably, 48 out of the 58 of these genes were also implicated in a previous proteomics-based approach to define a cancer matrisome^12^ and we further validated our signature at the proteomic level by examining transcript–protein correlations using matched BRCA samples from CPTAC^13^, wherein mostly positive correlations were observed for 37 out of the 49 C-ECM genes covered by both mass spectrometry and RNA-seq (Supplementary Figure 1D). Analysis using the CPTAC ovarian cancer data set also yielded similar correlations, with the caveat that only 24 C-ECM genes were represented in the mass spectrometric data set (Supplementary Figure 1E)^14^.

Upon summarisation using ssGSEA (single sample Gene Set Enrichment Analysis) scores^15,16^, these C-ECM genes show broad variation across tumour types (Fig. 1b, Supplementary Figure 1B,F). We then performed a Cox regression based on quartile-thresholded C-ECM scores with American Joint Committee on Cancer stage and tumour type as strata to examine the prognostic impact of this dysregulation; upregulated C-ECM genes were significantly associated with poor prognosis (Fig. 1c, d, hazard ratio (HR) = 1.73, *p* < 6.3e−7 for top versus bottom quartile, Cox regression, two sided) while downregulated C-ECM genes were not (Supplementary Figure 1G), suggesting that the variation we observed in C-ECM gene transcription is clinically relevant. We also tested for the independent prognostic capability of C-ECM genes by repeating this regression with non-ECM upregulated and downregulated gene score quartiles included as covariates and found that C-ECM-up scores were still prognostic (HR = 1.41, *p* = 0.003 for top versus bottom quartile, full model coefficients in Supplementary Table 3, Cox regression, two sided).

We also performed survival analyses where the quartiles of up-and-down scores were combined into single categories. This showed that cancers with the highest quartile of the up-score (Q4) and the lowest quartile of the down-score (Q1) had markedly increased risk of death (Supplementary Figure 1H, HR = 3.18, *p* < 8.5e−5, Cox regression, two-sided).

### C-ECM dysregulation is associated with the presence of CAFs

Given the previously identified role of distinct stromal cells in determining the composition and behaviour of the ECM^17^, we then attempted to infer the potential cell types associated with C-ECM transcriptional variation. Using a range of computational approaches, we examined whether changes in cellular composition, along with cell-type-specific transcriptional changes, could be associated with C-ECM gene dysregulation and found multiple indicators that C-ECM gene dysregulation originated in CAFs.

First, we sought to estimate whether there was a cancer-specific contribution to the C-ECM signature by examining correlations between C-ECM scores and tumour purity. This was motivated by the reasoning that if the C-ECM signature originated in the tumour epithelial compartment it would be positively associated with tumour cellularity.

**Fig. 2:**
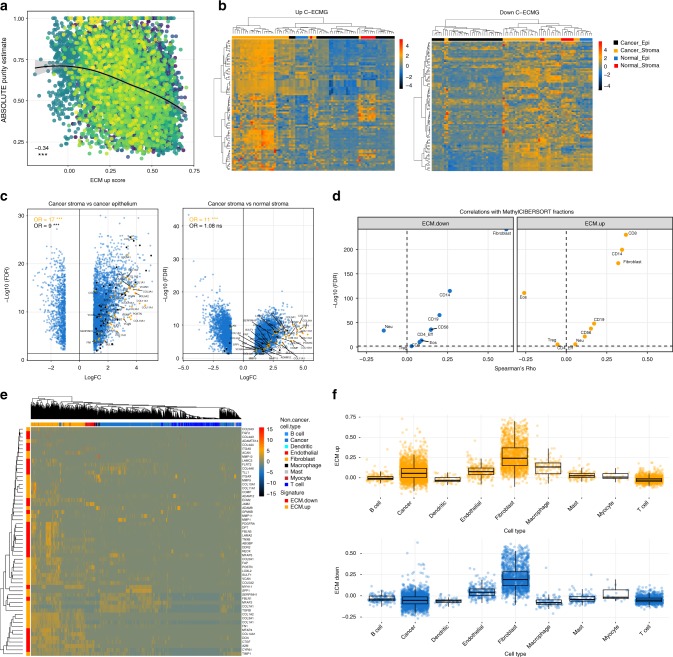
C-ECM transcription is associated with stroma, especially CAFs. **a** ABSOLUTE purity estimates are inversely correlated with C-ECM-up score, suggesting stromal origin; colours represent cancer types, number shows Spearman’s Rho (*n* = 8128). **b** Heatmaps of C-ECM-up and -down signatures projected onto microdissected epithelium and stroma from ovarian cancers. Rows show expression *z*-scores, samples are in columns. Annotation bars indicate tissue type. **c** Volcano plots show C-ECM genes (upregulated in orange, downregulated in black) differentially expressed between cancer stroma and epithelium and between cancer and normal stroma. **d** Volcano plots showing Spearman’s correlations between MethylCIBERSORT cell-type fractions and C-ECM scores. **e** Heatmap of C-ECM genes in single-cell HNSCC RNA-seq data (*n* = 5902). **f** CAFs show the highest expression of C-ECM genes relative to other cell types in single-cell HNSCC data. Numbers on scatterplots indicate Spearman’s Rho, asterisks indicate statistical significance. ***p < 0.001. On all volcano plots, *y* axis = −log_10_ fold change, *x* axis = test statistic/fold change/Spearman’s Rho. On volcano plots, all enrichment statistics are from Fisher’s Exact Tests

We found tumour purity estimated using ABSOLUTE, which jointly estimates cancer cell fractions of variants, purity and ploidy using copy-number data and whole-exome data^18^, was inversely correlated for both C-ECM-up and -down scores (Fig. 2a, S2A). This finding was additionally supported by inverse correlations between C-ECM scores based on the median variant allele frequency (VAF) (Rho = −0.17, for the up-score, and Rho = −0.36, down-score, *p* < 2.2e−16, Spearman’s correlation) and purity estimates independently computed using allele-specific copy number profiles using ASCAT^19^, downloaded from COSMIC^20^ (Rho = −0.28, up-score, and −0.3, down-score, *p* < 2.2e−16, Spearman’s correlation). The lower correlation of the median VAF with ECM scores is expected given the confounding influence of ploidy on the relationship between cancer cell fraction and VAF^21^.

The breakdown of correlations between C-ECM scores and purity estimates for different methods by tumour type have been presented in Supplementary Figure 2B, and collectively, these findings hinted at a stromal origin for C-ECM transcriptional changes.

We then leveraged multiple transcriptomic data sets that were separated into epithelial and stromal components by microdissection or cell sorting at various resolutions from ovarian cancer, head and neck cancer and colorectal cancer, as well as orthogonal pan-cancer DNA methylation-based deconvolution approaches in order to further evaluate the hypothesis of stromal origins and to identify specific cell types associated with the C-ECM dysregulation. First, projecting the C-ECM signature onto microdissected ovarian cancer stroma, matched epithelium and their normal counterparts^22^ (GSE40595) resulted in clustering by sample type with strong stromal expression (Fig. 2b). Additionally, probes differentially expressed between cancer epithelium and stroma were significantly enriched for both C-ECM-up and -down genes while differentially expressed probes between cancer and normal stroma were enriched in C-ECM-up genes alone (Fig. 2c). Altogether, these results suggest that our C-ECM-up signature comprises genes upregulated in the cancer stroma versus the normal stroma, while our C-ECM-down signature comprises genes upregulated in stroma (normal and cancer) versus epithelial tissue. In contrast, non-ECM genes in the pan-cancer cancer-versus-normal signature displayed weaker enrichment or epithelial associations in some cases (Supplementary Table 4).

Subsequently, DNA methylation-based deconvolution analysis using MethylCIBERSORT^23^ implicated CAFs, CD8 T cells and CD14 monocytes as directly correlated with C-ECM signature scores pan-cancer (Fig. 2d) in addition to confirming inverse associations with tumour purity (Supplementary Figure 2B). Importantly, C-ECM-up genes (ssGSEA scores) showed a positive correlation to the inferred CAF frequency in most TCGA cancer types (Supplementary Figure 2C). We also validated these DNA methylation-based inferences of cellular association using transcript levels of known marker genes for CYT (geometric mean of *GZMA* and *PRF1*), CD8 T cells (*CD8A* expression), CAFs (*ACTA2* expression) and monocytes (*CD14* expression) whereupon we noticed strong, consistent agreement (Supplementary Figure 2D).

Further validation of stromal and CAF association was then performed in three colorectal cancer microarray data sets (GSE39397 and GSE35144)^6,7^. In these data sets, C-ECM genes were strongly expressed in the stromal compartment and, when cell types were resolved further, mostly localised to fibroblast fractions (Supplementary Figure 2E). We also found that upon comparison of xenograft expression profiles (which retain just human tumour epithelium) with their matched primary tumour counterparts, the expression of C-ECM genes was completely ablated upon the loss of human tumour stroma (Supplementary Figure 2E).

Notably, the CAF specificity of C-ECM transcription was further indicated by the absence of expression in the leukocyte compartment in these data sets, suggesting that while monocyte enrichment and CD8 infiltration co-occur with C-ECM upregulation, they are not the source of C-ECM transcriptional changes (Supplementary Figure 2E).

Finally, as an ultimate test of a CAF origin, we examined a data set of single-cell transcriptomes from head and neck squamous cell carcinoma (HNSCC; GSE103322), which we selected owing to the large collection of CAFs (*n* = 1440) profiled in the study^24^. We found markedly higher expression of C-ECM genes in CAFs, which clustered together when the signature was projected onto the data set (Fig. 2e). In this data set, C-ECM-up and -down ssGSEA scores were significantly elevated in CAFs compared to other cell types (Fig. 2f), and this was also independently verified in an additional colorectal cancer single-cell RNA-seq data set, which contained a small number of CAFs (GSE81861, Supplementary Figure 2F)^25^. Finally, we also performed statistical testing for CAF association for each C-ECM gene in the HNSCC scRNA-seq data set and found that 45 genes were significantly overexpressed (mean difference >0, false discovery rate (FDR) < 0.05, Wilcoxon’s Rank Sum Test) and 10 genes were significantly underexpressed in CAFs compared to other cell types (Supplementary Figure 2G).

Collectively, these lines of evidence suggest that C-ECM profiles are generated mainly through the modulation of transcriptional profiles in CAFs as a general feature of malignancy.

### C-ECM dysregulation is correlated with immunological activity

Given that C-ECM scores correlate with CD8 T cells and CYT (Fig. 2d and Supplementary Figure 2D, and the fact that C-ECM-up scores are adversely prognostic despite the positive prognostic impact of CYT^9^, we postulated that the C-ECM-up score may be enriched in immunologically ‘hot’ tumours and may reflect an adaptive mechanism of immune evasion. Our subsequent analyses uncovered robust evidence for this association using multiple orthogonal approaches.

**Fig. 3:**
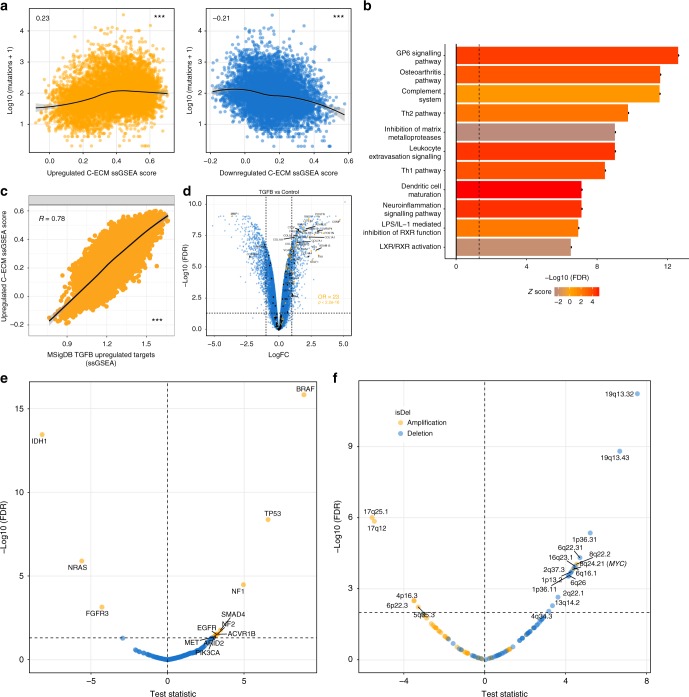
E-ECM scores are associated with immunologically hot tumours and TGF-β. **a** C-ECM-up and down scores are significantly associated with mutational burden across cancer types but in opposite directions. **b** Canonical pathway analysis shows activation of inflammatory/adaptive-immune pathways. **c** Correlation between C-ECM-up scores and enrichment for an aggregated list of upregulated non-C-ECM TGF-beta target genes across tissues and species from MSigDB. **d** Volcano plot showing enrichment for C-ECM genes in TGF-β-induced transcriptional changes in immortalised normal fibroblasts. Linear model *t*-statistics for candidate **e** mutational and **f** copy-number alterations associated with ECM-up ssGSEA scores, adjusted for tumour type, on volcano plots. Numbers on scatterplots indicate Spearman’s Rho, asterisks indicate statistical significance. ***p < 0.001. On all volcano plots, *y* axis = −log_10_ fold change, *x* axis = test statistic/fold change/Spearman’s Rho. On volcano plots, all enrichment statistics are from Fisher’s Exact Tests

Initially, we discovered that C-ECM-up score was positively correlated with mutational burden (Rho = 0.23, *p* < 2.2e−16) while the down-signature was negatively correlated (Rho = −0.21, *p* < 2.2e−16) (Fig. 3a). Similarly, C-ECM scores and Class I neoantigen burden also showed concordant associations (Rho = 0.21 and −0.21, *p* < 2.2e−16, Supplementary Figure 3A, Spearman’s correlation).

Then we tested for associations between C-ECM scores and microsatellite instability, an immunotherapy response biomarker per se^26^, and found significant associations (Supplementary Figure 3B).

Additionally, we assessed macrophage polarisation using CIBERSORT^27^ and found that the ECM-up signature was associated with a greater fraction of M1 (immunoactive) relative to M2 (immunosuppressive) macrophages (Supplementary Figure 3C). Finally, we found that multiple immune checkpoints, including *IDO1*, *B7-H3* and *PD-L2*, were overexpressed in samples in the top quartile of the C-ECM-up score distribution relative to bottom quartile cancers after adjusting for tumour type (2 fold change (FC), FDR < 0.01, Supplementary Data 1), indicating the upregulation of adaptive resistance mechanisms to immune-cell-mediated destruction (Supplementary Figure 3D). Moreover, these themes were broadly reinforced by ingenuity pathway analysis (IPA) canonical pathway analysis (Supplementary Data 2), which identified enrichment for inflammatory processes and adaptive immune responses enriched in samples in the top quartile of the C-ECM-up score (Fig. 3b).

### C-ECM dysregulation is linked to TGF-β activation

Next, since our data suggest that the C-ECM-up signature associated with the presence of CAFs and not with normal stroma (Fig. 2b, c), we endeavoured to find putative drivers responsible for this dysregulation. To do this, we divided samples into quartiles based on the C-ECM-up score and then performed linear modelling using limma-trend with cancer type as a covariate.

IPA causal network analysis, after restriction to candidate regulators which by themselves differentially expressed between C-ECM-up top and bottom quartiles, identified TGF-β as one of the most activated regulators (Supplementary Figure 3E) and upstream regulatory analysis further identified multiple *SMAD* transcription factors, *AP1* complex members that associate with SMADs^28^ and *SMARCA4*^29^ (Supplementary Figure 3F, Supplementary Data 3), all critical for TGF-β transcriptional responses as activated in c-ECM-up-high cancers.

Moreover, we also performed orthogonal analyses using TCGA reverse phase protein array (RPPA) data (*n* = 4278) and identified 13 differentially abundant peptides between upper and lower quartiles of the ECM-up score (FC > 1.3, FDR < 0.01, Supplementary Figure 3G). Notably, these included increased levels of fibronectin and PAI1, both ECM components, and a vast majority of these peptides have known prior associations in the literature with TGF-β (see Supplementary Table 5 for each reference), reinforcing the inference of activated TGF-β signalling.

Importantly, in our RNA-seq analyses, TGF-β is significantly overexpressed in upper quartile C-ECM-up cancers, along with multiple mediators of ECM deposition such as FGF family members (*FGF1*, *FGF18*), BMPs (*BMP1* and *BMP8A*) and the local sequestrators of TGF-β, *FBP1* and *LTBP1*. Further, we compiled a collection of TGF-β upregulated target genes across various tissues and across species from MSigDB (C2 collection) while excluding C-ECM genes and found a very strong correlation between C-ECM-up scores and this set (*R* = 0.78, *p* < 2.2e−16, Fig. 3c), suggesting TGF-β activation, and not only presence, is correlated with the C-ECM-up score. As a direct empirical test of the hypothesis that C-ECM-up genes are induced by TGF-β activation in fibroblasts, we compared the expression profiles of TGF-β-treated immortalised ovarian fibroblasts (GSE40266)^22^ versus untreated controls.

This revealed marked enrichment for C-ECM-up genes among differentially expressed genes (DEGs) (Fig. 3d, OR = 23, *p* < 2.2e−16, Fisher’s Exact Test), further strengthening the notion that C-ECM-up gene dysregulation is associated with TGF-β signalling in CAFs. However, C-ECM-down genes were not significantly enriched among downregulated genes following TGF-β stimulation, suggesting that C-ECM-up genes represent TGF-β activation in CAFs, while C-ECM-down genes represent more normal fibroblasts.

TGF-β is known to exert a multitude of effects in the tumour microenvironment; it is capable of stimulating fibrosis, inducing epithelial–mesenchymal transition and driving metastasis^30,31^. In contrast, it can also induce tumour-suppressive cytostasis^32^. We therefore tested whether distinct genomic adaptations disproportionately occurred in cancers with high C-ECM-up scores and whether these had known roles in TGF-β signalling.

We initially defined a set of candidate driver genes with evidence of positive selection in cancer and carried out linear modelling regressing the presence of nonsilent mutations against C-ECM-up score with cancer type as a covariate to identify genomic events that permitted adaptation to activation of the TGF-β pathway^33^. This uncovered multiple notable candidates, including *TP53*, *SMAD4*, *BRAF*, *ACVR1B* and *NF1/2* (Fig. 3e).

We also repeated the analysis using a compendium of 111 GISTIC^34^ copy-number peaks defined across TCGA and found 18 out of the 111 candidate peaks to be associated with C-ECM-up score, most notably *MYC* amplification at 8q24.3 (Fig. 3f). Most of these genomic events have been directly associated with TGF-β pathway signalling (Supplementary Table 6 lists the relevant literature).

### C-ECM dysregulation predicts failure of PD-1 blockade

Finally, we tested whether C-ECM dysregulation is an immune-evasion mechanism in the context of PD-1/PD-L1 blockade, where immunologically ‘hot’ tumours are associated with responses^35^. We did not include CTLA4-blockade data because of the small number of patients and qualitative differences in the biology of PD-1 and CTLA4 blockade^36^. Eligible data sets were required to contain RNA-seq data and matched mutation/class I major histocompatibility complex (MHC) counts from whole-exome data from pre-treatment biopsies.

**Fig. 4:**
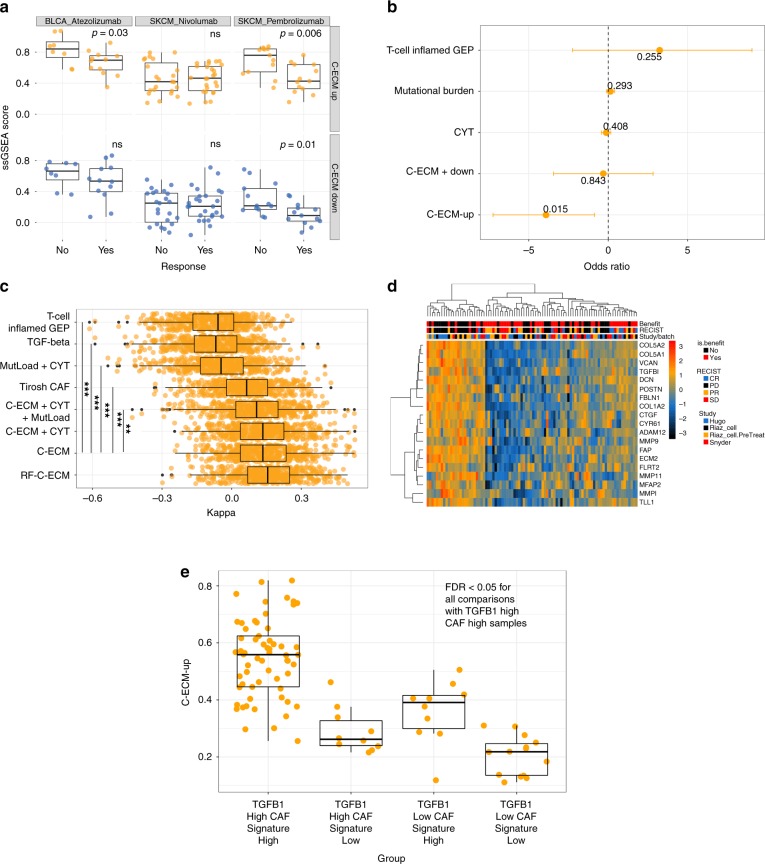
C-ECM scores predict failure of PD-1 blockade. **a** Boxplots showing distributions of C-ECM ssGSEA scores across multiple data sets of pretreatment biopsies from patients treated with PD-1 blockade. Non-responders = progressive disease (PD). *p* Values are from Wilcoxon’s Rank Sum Test. **b** Coefficients from pooled logistic regression analysis evaluating various predictors on PD-1-blockade response, error bars indicate 95% confidence intervals. **c** Boxplots of Cohen’s Kappa from 0.632 bootstrapping (500 resamples), showing that ECM-based models outperform other candidate biomarkers. Asterisks show *q*-values. **d** Heatmap showing C-ECM genes differentially expressed between ICB responders and nonresponders after controlling for study-specific variation. Rows show *z*-scores of expression (log2 CPM) and columns show samples; annotation ribbons display clinical information. **e** Boxplot showing C-ECM-up score distributions based on discretised categories of *TGFB1* expression and the Tirosh CAF signature. All comparisons with the TGFB1 high, CAF high group were significant at FDR < 0.05 (Wilcoxon’s Rank Sum Test)

In two out of three cohorts of PD-1-blockade-treated patients (two melanoma, one bladder)^37–39^, the C-ECM-up score was significantly higher in progressors (Fig. 4a, *p* < 0.05, Wilcoxon’s Rank Sum Test). This was also true in pooled logistic regression accounting for cancer type, CYT, mutational load, a T cell-inflamed signature^11^, cohort, antibody and prior anti-CTLA4 treatment (Fig. 4b).

Next, comparing prediction performance using logistic regression with 0.632 bootstrapping^40^ showed that models with C-ECM ssGSEA scores significantly outperformed those involving CYT, a T cell-inflamed signature and mutation load alone (Fig. 4c, S4A). This is notable given that the latter three factors have been proposed as biomarkers for patient stratification for immunotherapy. Moreover, the aggregate score is comparable to a random forest fit with individual C-ECM genes, suggesting that these genes may be largely co-regulated. Finally, restricted hypothesis testing using limma-trend found 19 C-ECM genes overexpressed at FDR < 0.1 (Fig. 4d) between responders and nonresponders, defining a practical signature for clinical application (Supplementary Figure 4B)

### C-ECM profiles independently predict PD-1-blockade failure

Our results were consistent with three models that we went on to further resolve. In one, *TGFB1/TGFB2* expression is associated with immunotherapy failure and there is no direct link with CAFs and the C-ECM programme. In the second, the presence of CAFs alone is sufficient and TGF-β-induced C-ECM gene expression does not add predictive value. In the final model, the C-ECM programme is activated by TGF-β in CAFs and is the causal determinant of immunotherapy failure.

To differentiate between these models, we applied the pooled logistic regression approach described earlier, as well as the 0.632 bootstrapping approach, to estimate model performance in an unbiased manner.

In pooled analyses, *TGFB1* and *TGFB2* expression by themselves did not predict immunotherapy failure (Supplementary Figure 4C, middle plot). A CAF signature derived from single-cell analyses of melanomas^10^ was used to estimate CAF abundance and this score was significantly associated (coefficient = −2.61, *p* = 0.03) (Supplementary Figure 4C, bottom plot), suggesting that CAF abundance plays a role.

However, this effect was stronger for the C-ECM-up score in the C-ECM-based model (coefficient = −4.03, *p* = 0.01), and when C-ECM scores, the CAF signature score and *TGFB1*/*2* expression are all included in a joint model, the CAF score loses significance while the C-ECM signature shows even stronger associations (coefficient = −8.47, *p* = 0.007) (Supplementary Figure 4C, top plot), consistent with the predictive value of CAFs being mediated through an association with the C-ECM-up signature. In accordance with these observations, in our machine learning evaluation, *TGFB1*/*TGFB2*-based models and the CAF-signature-based models are significantly outperformed by C-ECM-based models (Fig. 4c). Collectively, these results suggest that the C-ECM-up programme per se, and not simply CAF abundance or TGF-β activation in the tumour microenvironment in general, is associated with PD-1 blockade failure.

As a final test of the hypothesis that both TGF-β and CAFs are required for induction of the C-ECM signature, we binned samples from our immunotherapy data into low and high groups (first quartile versus all other quartiles) for TGFB1 expression and the Tirosh CAF ssGSEA score. We predicted that CAF-high TGFB-high cancers would display the highest C-ECM-up score, that CAF-low TGFB-low cancers would display the lowest enrichment, while the other two combinations would display significantly lower expression than CAF-high TGFB-high cancers because both factors are necessary but insufficient alone. Statistical comparisons (pairwise Mann–Whitney tests, FDR correction) confirmed our prediction (Fig. 4e).

## Discussion

In this study, we have identified a pan-cancer role for ECM dysregulation. Using a range of approaches, we show that ECM dysregulation is associated with the presence of CAFs and the activity of TGF-β, among other regulators. Our model fits a scenario where the C-ECM-down signature represents normal/normal-like fibroblasts, whereas the TGF-β-driven C-ECM-up signature identifies a poor prognosis CAF activation phenotype that is upregulated pan-cancer. We further show that tumours that are otherwise 'immunologically hot' tend to display higher levels of the C-ECM-up programme and possess genomic alterations that may minimise the fitness costs of the high levels of TGF-β signalling required to sustain the C-ECM-up programme. These statistical associations serve as suggestive early evidence supporting the continued experimental investigation of TGF-β in immune modulation across tissue types.

Given this, the depletion of CAFs may be a potential approach to enhance checkpoint blockade; however, in some cases, CAF depletion per se is paradoxically associated with worse outcomes^41^. This suggests approaches that seek to normalise the aberrant transcriptome in fibroblasts, possibly through TGF-β blockade, are likely to offer a more promising route to boost the efficacy of checkpoint blockade. Consistent with this, recent preclinical studies have uncovered evidence that simultaneous targeting of both TGF-β and PD-L1 can result in markedly better tumour control in multiple mouse models^42^. Moreover, recent studies using genetically reconstituted murine colorectal cancers^43^, and those based on transcriptional analyses of bladder cancers treated with PD-L1 blockade^7^, have arrived at similar conclusions and have demonstrated that TGF-β blockade can markedly potentiate antitumour immunity by modulating CAF-mediated T cell-exclusion phenotypes.

Our pan-cancer analyses establish the presence of a TGF-β-associated C-ECM programme that is correlated with immunotherapy failure and indicate the existence of subsets of C-ECM-high cancers independent of tissue type. Relevantly, prior work has suggested that negative selection in cancer is generally weak as a pan-cancer phenomenon, suggesting that the depletion of immunogenic mutations through immunoediting is weak^33^. The widespread occurrence of the C-ECM programme across cancer types may help explain, at least partially, why this is the case.

Our results suggest that tumours with elevated C-ECM programme across cancer types may likely benefit from combination immunotherapy with PD-1 blockade and TGF-β blockade to restore immune control of tumours. Future tissue-agnostic, C-ECM biomarker-guided basket trials are necessary to validate this hypothesis.

Finally, our findings linking TGF-β activity, CAFs, C-ECM signature and immunosuppression of otherwise immune ‘hot’ tumours are associative and further experiments would be required to prove a causal link.

## Methods

### General statistical procedures

All tests were two sided and were performed for unmatched samples measured once.

### Analysis of ECM signatures in cancer

RNA-seq data were obtained in the form of RSEM estimates from SAGE Synapse. This was reduced to 15 cancer types that had >10 normal samples available and low expressed genes were filtered (average <1 CPM).

Limma-trend^44^ was then used to compute DEGs between normal and cancer samples while controlling for cancer type at thresholds of FDR < 0.01, 2FC. Controlling for the cancer type was explicitly used in order to minimise the effects of class imbalance and also to derive a picture of ECM gene dysregulation independent of cancer type.

A reference gene set of extracellular matrix genes was derived using the gene ontology term 'extracellular matrix' (GO:0031032) biological process category (used by UniprotKB) and downloaded from geneontology.org. Gene ontology terms and enrichments in overlap were computed using Fisher’s Exact Test.

Significant upregulated and downregulated ECM genes were used to define gene sets whose expression was summarised using ssGSEA^15^ for each sample. Clinical data were then derived from SAGE synapse for a large subset of these tumours (Accession: syn7343873) and ECM-up and -down scores were divided into quartiles for categorisation. Validation at the protein level was performed using Spearman’s Rank Correlation between RNA-seq and mass spectrometry for matched BRCA and OVCA samples profiled by TCGA and CPTAC, with the mass spectrometric data derived from the corresponding publications.

A stratified Cox regression model was of the form Survival ~ ECM-up-quartile + ECM-down-quartile + strata(cancer type) + strata(stage) was fit to estimate impact on prognosis. Similarly, a combined analysis was performed by fusing ECM-up and -down quartile categories.

### Microenvironmental analyses of ECM dysfunction

ABSOLUTE^18^ was used to derive purity and ploidy estimates for *n* = cancers. ECM scores were tested for association with purity using Spearman’s Rank Correlations. This analysis was then repeated using purity estimates from ASCAT^19^ derived from COSMIC and the median VAF of mutations was obtained from the MC3 MAF (Synapse:syn7214402)^45^.

An ovarian cancer data set was then obtained from GEO (GSE40595) and normalised using fRMA^46^. C-ECM genes were then projected onto the data set to visualise clustering by phenotype. Significant probes were then identified for cancer epithelium versus cancer stroma and cancer stroma versus cancer epithelium using limma. Fisher’s Exact Tests were used to test for enrichment of ECM signatures among DEGs (defined using limma).

Cellular deconvolution analyses were carried out using estimated cellular fractions produced using MethylCIBERSORT using Spearman’s Rank Correlations between the ECM-up and -down scores. These findings were validated using gene expression of associated markers (*ACTA2*, *CD14*, *CD8A* and CYT) using Spearman’s Rank Correlation. Validation of CAF association was performed in single-cell RNA-seq data from HNSCC, selected owing to the availability of large numbers of CAFs (*n* = 1440) (GSE103322) by projecting ECM signatures onto log2(TPM + 1) transformed matrices. Data were obtained in a TPM matrix from GEO and the cell-type allocations were taken from the corresponding publication, wherein known cellular markers were used to define populations. ssGSEA scores were computed and Mann–Whitney tests were used to test for differential expression.

Additional validation was performed using count data for colorectal cancer single cells from GSE81861. Flow-sorted samples and colorectal cancer xenograft data were obtained from GEO data sets (GSE39397 and GSE35144)^6,7^ and heatmapping was used to visualise associations between C-ECM gene expression and cell type.

### Immunological correlates of ECM dysfunction

HLA type for MHC class I alleles was retrieved from The Cancer Immunome Atlas^47^. Topiary (https://github.com/openvax/topiary) was then used in combination with the MC3 set of TCGA mutation calls (Synapse:syn7214402) to define variants and potential neoantigens, using nonamers and thresholds of binding affinity <500 nM. Mutation loads were also computed for the same set of samples.

Spearman’s correlation and negative binomial models were used to test for associations between these genomic counts and ECM scores after adjusting for cancer type. M1/M2 macrophage fractions were computed using CIBERSORT^27^.

The expression of immune checkpoints was computed as part of whole-transcriptome analyses from differential expression between the top and bottom quartiles of the ECM-up score, adjusting for cancer type. Mismatch repair analyses were carried out using TCGA-allocated status based on Sanger sequencing published previously^48^.

### Multiplatform correlates of ECM dysfunction

For RNA-seq and RPPA data, limma-trend was used to compute differentially regulated genes/proteins at thresholds of 2FC, FDR < 0.01 and 1.3FC, FDR < 0.01 by comparing samples in the top and bottom quartiles of ECM-up score, respectively.

IPA was used to perform a core pathway analysis using interactions experimentally verified in human tissues in general. Causal network analysis results were restricted to those themselves differentially expressed, while upstream regulatory analyses were not.

For mutation analyses, we retrieved a previously published catalogue of driver mutations^33^ (positively selected) in cancer and carried out linear regression against ECM-up score with cancer type for a covariate. For copy-number data, we generated GISTIC^34^ calls pan-cancer at intensity thresholds of 0.3 and FDR < 0.01. Events for each GISTIC peak were dichotomised into altered/wild type and were regressed against ECM-up scores using a linear model with cancer type as covariate.

### Analysis of immunotherapy data sets

We assembled RNA-seq, mutation and neoantigen burden data from multiple immunotherapy data sets and standardised the expression data to consider transcripts quantified in all data sets. Then we computed ssGSEA scores for the C-ECM-up and -down scores for each data set and examined association with response (partial response/complete response/stable disease versus no response) by Response Evaluation Criteria in Solid Tumours criteria using Mann–Whitney tests. A pooled logistic regression was conducted using the C-ECM scores from the three data sets with cancer type and treatment as covariates.

Finally, 100 iterations of 0.632 bootstrapping^40^ were carried out to evaluate the performance of logistic regression and Cohen’s unweighted Kappa was used to evaluate model performance. For Random Forest-based analyses, we first subtracted study-specific effects using a linear model via the removeBatchEffects function in the limma R package. Mann–Whitney tests were used to compare model performance across resamples. The models fit were C-ECM + CYT, C-ECM alone, TGFB1 alone, Mutational load + CYT and C-ECM, CYT and mutational load, a CAF signature derived from Tirosh et al.^10^ and an interferon-induced signature^11^ (and in each case, the study/cohort was included as a covariate). Limma-trend was used to identify ECM genes differentially expressed between nonresponders and responders using restricted hypothesis testing at FDR < 0.1. Finally, for analysis of mechanistic hypotheses surrounding the relative contributions of CAFs, TGF-β and the C-ECM-up score to immunotherapy failure, we fit the following logistic regression models. (i) Response ~ TGFB1 + TGFB2 + log2(MutLoad) + T cell-inflamed GEP score + Cohort, (ii) Response ~ Tirosh CAF signature score + log2(MutLoad) + T cell-inflamed GEP score + Cohort, (iii) Response ~ C-ECM-up score + Tirosh CAF signature score + TGFB1 + TGFB2 + log2(MutLoad) + T cell-inflamed GEP score + Cohort. To test whether both TGFB1 and CAFs were required for activation of the C-ECM-up signature, we defined the lowest quartile of the Tirosh CAF signature score, and the lowest quartile of TGFB1 expression, as low and the rest as high. Pairwise Mann–Whitney tests were computed between the four groups produced thus with Benjamini–Hochberg correction for FDR.

### Code availability

Knit HTML R markdowns of the code used to generate the results are available on Zenodo at 10.5281/zenodo.1410639.

### Reporting Summary

Further information on research design is available in the Nature Research Reporting Summary linked to this article.

## Electronic supplementary material

Supplementary Information

Description of Additional Supplementary Files

Supplementary Data 1

Supplementary Data 2

Supplementary Data 3

Reporting Summary

## Data Availability

TCGA data used in this paper are available from SAGE Synapse (https://www.synapse.org) at accessions syn7343873, syn4311114, syn4303551 and syn7214402. Single-cell RNA-seq and gene expression microarray data used in this study are available at GEO (https://www.ncbi.nlm.nih.gov/geo/) accessions GSE40595, GSE103322, GSE39397, GSE35144 and GSE81861. RData files of intermediate processed objects are available upon reasonable request from the authors.
