# Supplementary material for: TGF-β-associated extracellular matrix genes link cancer-associated fibroblasts to immune evasion and immunotherapy failure
Source: Nat Commun. 2018 Nov 8;9:4692. doi: 10.1038/s41467-018-06654-8 (PMC6224529; doi:10.1038/s41467-018-06654-8)
Supplement: Supplementary file 1 — Supplementary Information [file 41467_2018_6654_MOESM1_ESM.pdf]

Supplementary Information for Chakravarthy et al, TGF-B associated extracellular matrix genes link cancer associated fibroblasts to immune evasion and immunotherapy failure.

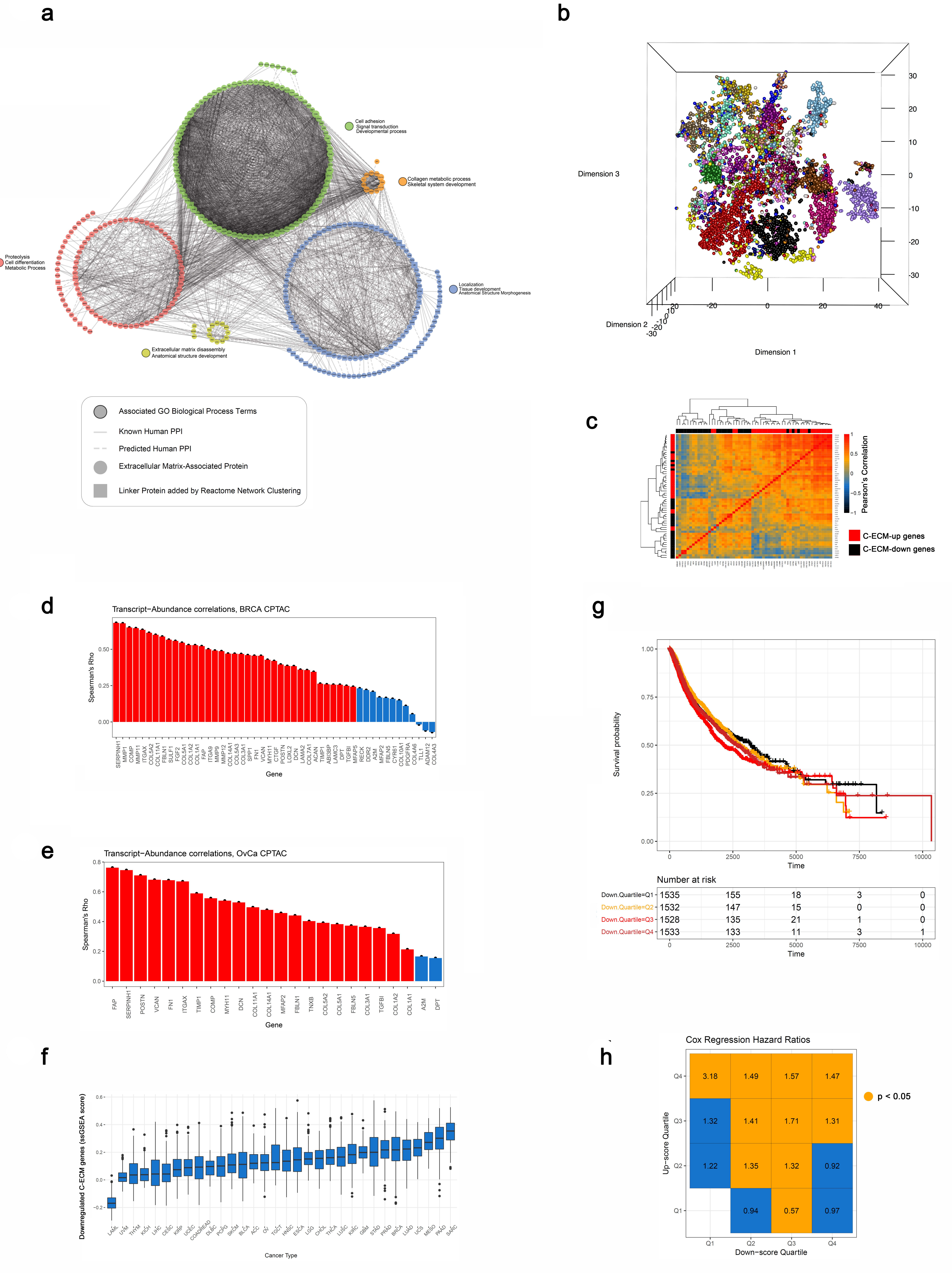

Supplementary Figure 1: a. Reactome ontology analysis for genes in the ECM gene set. b. tSNE visualisation of all ECM genes, suggesting variation across cancer types, which led us to focus on developing a pan-cancer signature after accounting for tumour type. c. Heatmap of inter-gene Pearson correlations between C-ECM genes. d. and e.Validation of C-ECM signature at the protein level in matched proteome/transcriptome data from TCGA/CPTAC for Breast Cancer. Each bar represents a gene and the y-axis represents the Spearman's Rho between transcript and peptide levels. Red indicates correlations at FDR < 0.05 f. Distribution of C-ECM-Down-scores by tumour type on a tSNE plot, g. K-M curves based on quartiles of C-ECM-down scores. h. Hazard ratios from Cox regression modelling based on combinations of up-score quartile and down-score quartile. Associations significant at p < 0.05 are coloured in orange. For boxplots, box-ends represent upper and lower-quartiles, centerlines represent medians, and whiskers represent 1.5x interquartile range (IQR).

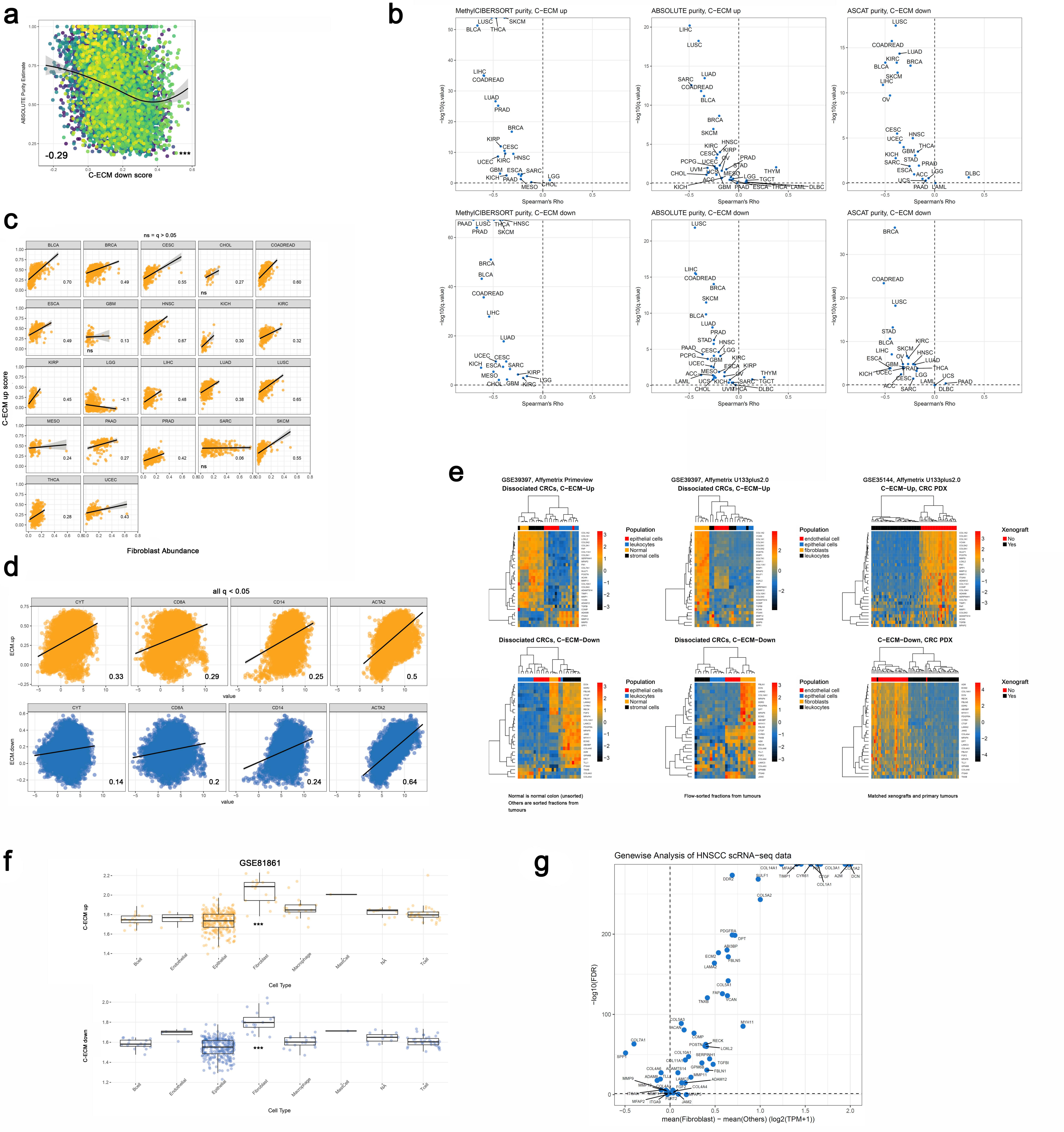

Supplementary Figure 2: a. Inverse correlation between ABSOLUTE purity estimate and C-ECM-down score. b. Correlation breakdown by tumour type based on ABSOLUTE, ASCAT and MethylCIBERSORT between purity estimates and C-ECM ssGSEA scores. All plots show correlation coefficient on the x-axis and -log10(FDR) on the y-axis. c. Correlations between C-ECM-up score and MethylCIBERSORT fibroblast estimates across cancer types. d. Plots showing correlations between marker gene expression and C-ECM scores, confirming correlations inferred using MethylCIBERSORT. e. Heatmaps showing stromal/fibroblast association of C-ECM genes in additional colorectal cancer microarray datasets. f. Validation of CAF-origin for C-ECM scores in an independent scRNAseq dataset from colorectal cancers. g. Volcano-plot showing mean difference in log2(TPM+1) for C-ECM genes between CAFs and other cell-types in HNSCC scRNA-seq data (Mann Whitney Test). Y axis represents -log10(FDR). For boxplots, box-ends represent upper and lower-quartiles, centerlines represent medians, and whiskers represent 1.5x interquartile range (IQR)

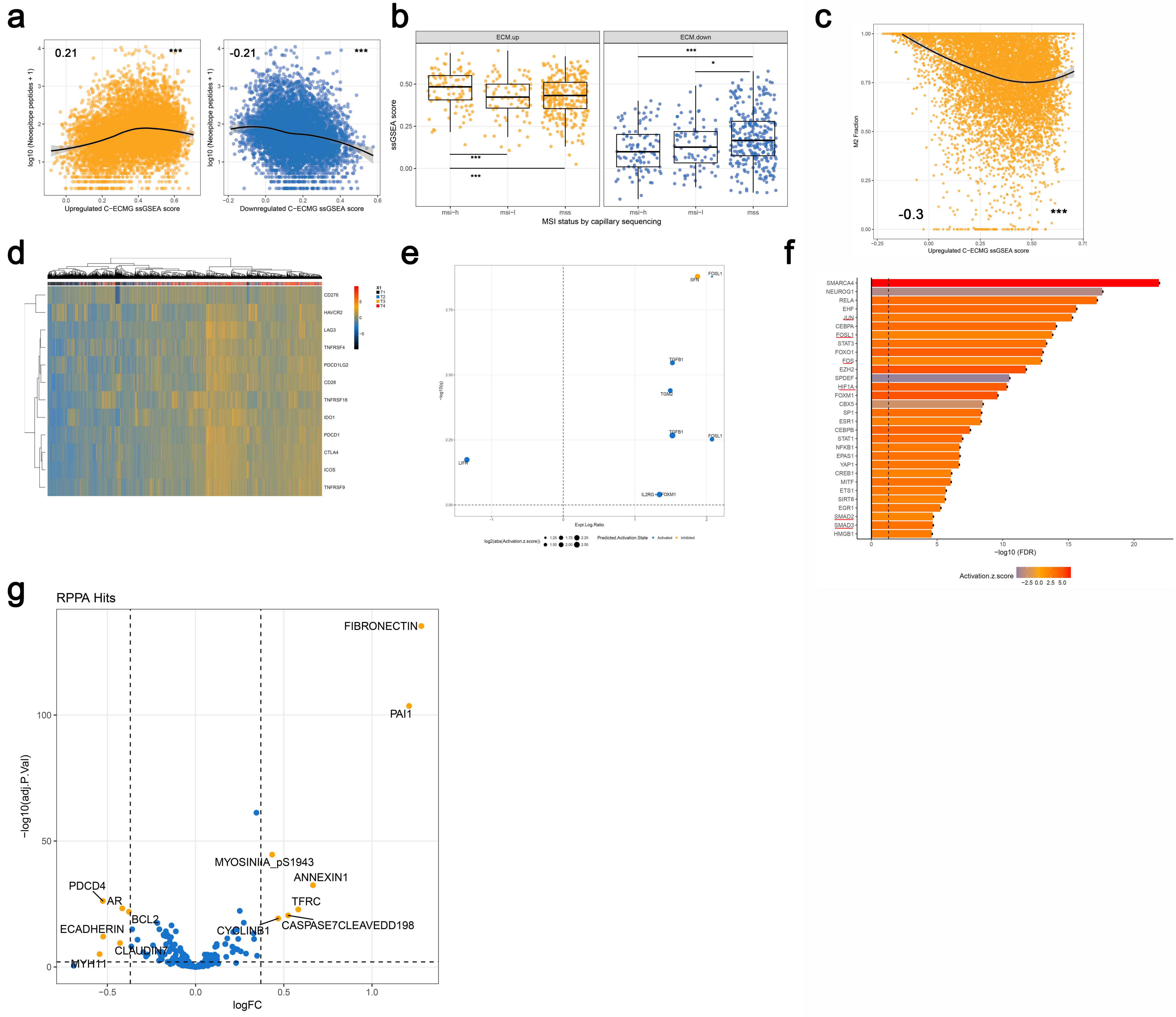

Supplementary Figure 3: a. Correlations between numbers of Class I MHC neoepitopes estimated using Topiary and C-ECM scores. b. C-ECM-scores show associations with microsatellite instability as inferred using capillary sequencing. c. Relationship between M1/M2 fraction and C-ECM-up score. d. Heatmap of immune checkpoints (ligands and receptors) different between cancers in upper and lower quartiles of C-ECM-up score. e. Volcano plot showing results from Causal Network Analysis of genes differentially expressed between C-ECM-up score upper and lower quartile cancers. f. Results of upstream regulatory analysis (y axis = regulator, x axis = -log10(FDR)) based on genes differentially expressed between C-ECM-up score upper and lower quartile cancers. Barplot shows breakdown of hot and cold tumours by C-ECM-up score quartile. g. Volcano plot showing differentially abundant peptides/epitopes between C-ECM-up score upper and lower quartile cancers.

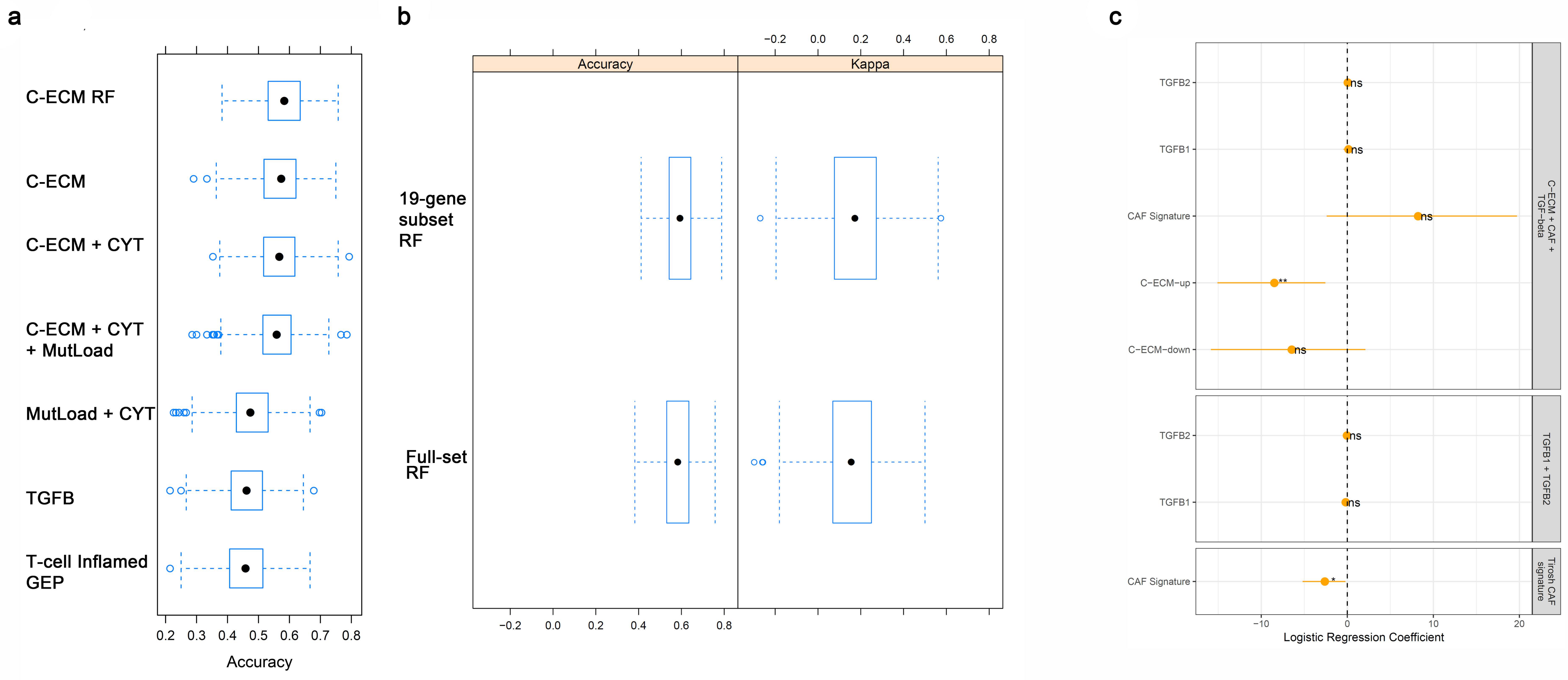

Supplementary Figure 4. a. Boxplots showing breakdown of performance using Accuracy instead of Cohen's Kappa for many of the models in Figure 4C. b.Boxplots showing Cohen's Kappa and Accuracy for random forests trained using all C-ECM genes and those trained with the subset differentially expressed between immunotherapy responders(CR/PR/SD) and nonresponders(PD). c. Logistic Regression Model coefficients and p.values for models regressing immunotherapy response against a CAF signature alone, TGFB1/2 expression alone, and a model consisting of C-ECM signatures, a CAF signature, and TGFB1/2 expression alone. For boxplots, box-ends represent upper and lower-quartiles, centrelines or central points represent medians, and whiskers represent 1.5x interquartile range (IQR). Error bars represent 95% confidence intervals for logistic regression plots.

Supplementary Table 1

ITGA2	ERO1A	SH3PXD2A	FBN2
COL5A1	SFRP2	NF1	FBN1
LAMC3	ELN	COL4A4	HAPLN2
DCN	CTSS	C6orf15	BMP1
LAMA3	KDR	COL9A1	MFAP5
CPB2	KLK2	CST3	SERPINF2
ITGA11	SMOC2	TIMP1	ITGA5
PRSS2	NDNF	AGT	COL4A5
CYP1B1	FBLN5	ADAMTS14	ICAM3
COL6A3	VPS33B	LAMC1	CTSL
COL6A2	OPTC	TGFBI	FLRT2
COL6A1	SMAD3	POMT1	KLK7
COL11A1	SPINT1	ITGAV	ICAM1
FSCN1	PLG	SOX9	CAPNS2
CTRB2	PRSS1	CYR61	ADAMTS5
HSPG2	NR2E1	EGFLAM	RGCC
FAP	CREB3L1	TLL2	ITGAD
PLOD3	ICAM4	SERPINB5	COL4A3
GREM1	ITGB1	ADAMTSL4	DMP1
COL4A1	MMP14	EXOC8	FGG
COL3A1	LRP1	PDPN	FGB
COL2A1	P4HA1	CSGALNACT1	FGA
COL1A1	COL7A1	TNFRSF11B	ITGA1
FERMT1	ITGA4	PDGFRA	ADAMTS3
NPHS1	VCAN	HTRA1	CDH1
LAMA2	ADAM10	MMP16	WT1
THBS1	ABI3BP	MMP15	MMP10
LAMB1	DPT	LOXL3	MMP7
VCAM1	MELTF	COL10A1	
BCAN	DSPP	POSTN	
CTRB1	CD47	ETS1	
TNXA	NOX1	TIMP2	
ABL1	COL8A1	CAPNS1	
BCL3	HSD17B12	SCX	
DPP4	PTK2	TMPRSS6	
PRDX4	URS000024463E_9606	ITGA7	
OLFML2A	CRISPLD2	CHADL	
SPARC	PDGFB	NOTCH1	
FN1	LOX	MYF5	
CCDC80	HAS2	ITGB8	
MMP11	MMP19	ITGB7	
LOXL1	COL1A2	ADAMTSL2	
DDR1	SPOCK2	ITGA3	

CAPN2	COL16A1	FURIN	
TLL1	ITGA8	WNT3A	
CAPN1	IBSP	TNC	
MPV17	BGN	KAZALD1	
ANXA2	SCUBE3	LUM	
PDGFA	TCF15	NPNT	
CMA1	SERPINH1	VIT	
VTN	TTR	TNF	
CTSV	CD44	COL27A1	
MATN3	JAM2	WASHC1	
LAMA5	LCP1	TGFBR1	
ITGA10	VIPAS39	AGRN	
COL8A2	CFLAR	ADAM8	
COL18A1	GAS6	NID1	
ADAMTS20	ITGB2	SH3PXD2B	
ADAM19	MMP1	GPM6B	
LAMA4	KLKB1	MADCAM1	
RECK	APP	ADAM15	
COL5A2	FOXC1	JAM3	
MMP20	B4GALT1	TNR	
SPINK5	MYH11	COL14A1	
RIC8A	PHLDB2	ERO1B	
DDR2	LAMB2	TGFB1	
SPINT2	NFKB2	DAG1	
COMP	CTSG	RAMP2	
CTGF	ACAN	ITGB3	
CLASP2	ITGB6	BSG	
SERAC1	LAMB3	MYO1E	
FLOT1	ITGA9	FOXF2	
CARMIL2	TGFB2	FOXF1	
ICAM5	ELANE	LAMA1	
ENG	NPHP3	COL11A2	
ATXN1L	CLASP1	COL9A2	
MMP8	MFAP2	EGFL6	
SERPINE1	FBLN1	LAMC2	
FMOD	KIF9	PHLDB1	
NID2	PECAM1	ITGA2B	
FGF2	MATN1	ELF3	
ANTXR1	ITGAM	MMP9	
VWA1	HPN	COL4A2	
THSD4	VWF	MMP2	
TNXB	MMP12	MMP3	
ITGAX	COL12A1	HAPLN1	
ITGAL	FOXC2	MFAP4	

SPP1	HPSE2	SCUBE1	
COL19A1	A2M	ECM2	
GFOD2	TPSAB1	SULF1	
ATP7A	F11R	SULF2	
NOXO1	COL13A1	HAS3	
DNAJB6	HAS1	ITGB4	
ADAMTS4	CTSK	ITGA6	
ADAM12	ITGAE	PXDN	
FGFR4	APBB2	MMP13	
MATN4	LOXL2	ICAM2	
ADAMTS2	ITGB5	NCAN	
COL5A3	ERCC2	COL9A3	
IHH	MPZL3	COL4A6	

Supplementary Table 2

logFC	AveExpr	T	P.Value	adj.P.Val	B	ID	is.ECM	is.upSig	is.downSig	is.sig
3.88317	5.347023	53.7002	0	0	1202.634	MMP11	Yes	Yes	No	Yes
-2.85059	4.141546	-41.3598	0	0	758.196	TNXB	Yes	No	Yes	Yes
-3.66323	2.279534	-40.2158	0	0	720.3502	DPT	Yes	No	Yes	Yes
2.351311	1.420181	37.15318	1.9E-276	1.1E-274	622.4141	ADAMTS14	Yes	Yes	No	Yes
-1.86451	2.077423	-34.9136	9.3E-247	4.2E-245	554.1347	GPM6B	Yes	No	Yes	Yes
1.097931	7.643832	34.57119	2.5E-242	1E-240	543.9592	SERPINH1	Yes	Yes	No	Yes
-2.73276	4.770041	-34.3025	6.9E-239	2.8E-237	536.0226	MFAP4	Yes	No	Yes	Yes
3.506895	2.276359	33.83967	5.5E-233	2.1E-231	522.4597	COL10A1	Yes	Yes	No	Yes
-2.55413	7.175302	-33.5345	3.9E-229	1.4E-227	513.5883	DCN	Yes	No	Yes	Yes
3.941206	1.738904	33.38066	3.4E-227	1.2E-225	509.1396	COL11A1	Yes	Yes	No	Yes
-1.74214	4.897319	-33.2138	4.2E-225	1.4E-223	504.3316	FBLN5	Yes	No	Yes	Yes
-1.70129	2.060619	-33.0972	1.2E-223	4E-222	500.9813	JAM2	Yes	No	Yes	Yes
-2.78801	6.206148	-32.298	9.2E-214	2.7E-212	478.2518	MYH11	Yes	No	Yes	Yes

-2.29348	3.495044	-30.0332	9.5E-187	2.1E-185	416.1297	ABI3BP	Yes	No	Yes	Yes
3.123632	2.542582	29.83522	1.9E-184	3.9E-183	410.8634	MMP1	Yes	Yes	No	Yes
2.062079	10.00793	29.62313	5.1E-182	1E-180	405.2528	COL1A1	Yes	Yes	No	Yes
1.412455	5.171597	29.19496	3.9E-177	7.5E-176	394.0214	LOXL2	Yes	Yes	No	Yes
2.410589	4.505334	29.08286	7.3E-176	1.4E-174	391.1024	MMP9	Yes	Yes	No	Yes
2.030705	3.288621	28.66273	3.9E-171	6.8E-170	380.2407	ADAM12	Yes	Yes	No	Yes
2.050433	1.346764	27.73949	6E-161	9.1E-160	356.8154	ACAN	Yes	Yes	No	Yes
-1.16688	3.105598	-27.7392	6E-161	9.2E-160	356.8091	RECK	Yes	No	Yes	Yes
-1.88248	4.429109	-26.7653	1.7E-150	2.3E-149	332.7693	PDGFRA	Yes	No	Yes	Yes
2.313136	6.861916	26.74155	3.1E-150	4E-149	332.1926	SPP1	Yes	Yes	No	Yes
1.913689	3.088895	26.44005	4.6E-147	5.8E-146	324.8983	FAP	Yes	Yes	No	Yes
-1.7602	3.490671	-25.7628	4.8E-140	5.5E-139	308.7623	LAMA2	Yes	No	Yes	Yes
-1.4027	6.853313	-25.6061	1.9E-138	2.2E-137	305.079	CYR61	Yes	No	Yes	Yes
-1.59279	2.630781	-25.4814	3.6E-137	3.9E-136	302.1587	FGF2	Yes	No	Yes	Yes
-2.3416	1.019502	-25.316	1.7E-135	1.8E-134	298.3083	COL4A6	Yes	No	Yes	Yes
1.304281	3.743698	25.23711	1.1E-134	1.1E-133	296.4773	ADAM8	Yes	Yes	No	Yes
1.375429	6.626083	24.56744	5E-128	4.9E-127	281.135	COL5A2	Yes	Yes	No	Yes
-1.65321	1.996744	-23.6674	2.7E-119	2.4E-118	261.0662	DDR2	Yes	No	Yes	Yes
1.143959	8.043102	23.42333	5.7E-117	4.8E-116	255.7339	TIMP1	Yes	Yes	No	Yes
-1.24618	3.160757	-23.4068	8.2E-117	6.8E-116	255.3734	ECM2	Yes	No	Yes	Yes
-1.60428	5.105669	-23.2911	1E-115	8.3E-115	252.8642	COL14A1	Yes	No	Yes	Yes
-1.06434	9.098173	-22.9356	2.1E-112	1.7E-111	245.22	A2M	Yes	No	Yes	Yes

-1.79006	0.703145	-22.3599	4.2E-107	3.1E-106	233.0575	COL4A3	Yes	No	Yes	Yes
2.225736	1.067224	22.00206	7.2E-104	5.1E-103	225.6333	MMP12	Yes	Yes	No	Yes
-1.35091	6.670897	-21.5074	1.8E-99	1.19E-98	215.5428	FBLN1	Yes	No	Yes	Yes
-1.29586	2.615176	-21.3485	4.38E-98	2.88E-97	212.3437	ITGA9	Yes	No	Yes	Yes
1.173681	3.796073	21.25901	2.64E-97	1.72E-96	210.5514	ITGAX	Yes	Yes	No	Yes
1.282316	6.912391	20.5547	2.9E-91	1.73E-90	196.678	COL5A1	Yes	Yes	No	Yes
-1.38438	2.324623	-20.3441	1.71E-89	9.95E-89	192.61	COL4A4	Yes	No	Yes	Yes
1.67356	3.641377	19.59627	2.47E-83	1.32E-82	178.4682	COL7A1	Yes	Yes	No	Yes
1.147605	4.091371	19.52256	9.73E-83	5.16E-82	177.1	COL5A3	Yes	Yes	No	Yes
-1.07176	7.224462	-19.1368	1.19E-79	6.03E-79	170.0161	CTGF	Yes	No	Yes	Yes
-1.29132	2.78163	-18.9148	6.68E-78	3.31E-77	165.9967	FLRT2	Yes	No	Yes	Yes
1.073881	7.431812	18.60035	1.88E-75	8.99E-75	160.3755	TGFBI	Yes	Yes	No	Yes
2.195006	2.412926	18.58269	2.57E-75	1.23E-74	160.0624	COMP	Yes	Yes	No	Yes
1.25434	3.84291	18.57932	2.73E-75	1.3E-74	160.0027	MFAP2	Yes	Yes	No	Yes
1.181232	6.547171	17.14779	1.26E-64	5.13E-64	135.5301	VCAN	Yes	Yes	No	Yes
1.072322	9.702256	16.99539	1.55E-63	6.21E-63	133.0309	COL1A2	Yes	Yes	No	Yes
-1.14272	0.78539	-16.9885	1.73E-63	6.95E-63	132.9177	TLL1	Yes	No	Yes	Yes
1.126595	9.62294	16.88405	9.55E-63	3.77E-62	131.2179	COL3A1	Yes	Yes	No	Yes
1.075476	6.315995	16.21222	4.44E-58	1.63E-57	120.5132	SULF1	Yes	Yes	No	Yes
1.279737	6.446411	15.88976	6.69E-56	2.37E-55	115.5185	POSTN	Yes	Yes	No	Yes
1.127548	10.36994	15.79289	2.96E-55	1.04E-54	114.0363	FN1	Yes	Yes	No	Yes
-1.09919	2.354247	-15.4895	2.97E-53	1.01E-52	109.4489	LAMC3	Yes	No	Yes	Yes
-1.3285	1.720066	-14.0038	5.6E-44	1.65E-43	88.19267	MFAP5	Yes	No	Yes	Yes

Supplementary Table 3

Term	coef	HR	Std.Error	Z	p.value	Significance
**ECM.up.qQ2**	0.19453	1.21474	0.09347	2.081	0.03741	*
**ECM.up.qQ3**	0.30455	1.35601	0.1058	2.879	0.00399	**
**ECM.up.qQ4**	0.34764	1.41572	0.11941	2.911	0.0036	**
**ECM.down.qQ2**	0.01757	1.01773	0.0789	0.223	0.82376	
**ECM.down.qQ3**	0.0619	1.06385	0.09473	0.653	0.5135	
**ECM.down.qQ4**	-0.02759	0.97278	0.12184	-0.226	0.82084	
**NonECM.up.Q2**	0.4312	1.53911	0.10313	4.181	2.90E-05	***
**NonECM.up.Q3**	0.77113	2.16222	0.12653	6.094	1.10E-09	***
**NonECM.up.Q4**	0.79609	2.21685	0.14612	5.448	5.09E-08	***
**NonECM.down.Q2**	0.04464	1.04565	0.07919	0.564	0.57294	
**NonECM.down.Q3**	0.10356	1.10912	0.10961	0.945	0.34473	
**NonECM.down.Q4**	0.20076	1.22233	0.14335	1.401	0.16136	

Supplementary Table 4

Microarray / Cancer vs Normal	ECM-up	ECM-down	Non-ECM-up	Non-ECM-down
**Stroma-vs-Epithelium-up**	17.89	9.13	0.7	4.18
**Cancer-stroma-vs-normal-stroma-up**	11.49	1.08	1.74	0.84

blue = p > 0.05

Supplementary Table 5

Antibody	Status	Phenotype	Reference
Fibronectin	Up	TGF-β induces Fibronectin synthesis through a transcriptional mechanism	[1]
PAI1	Up	TGF-β induces expression through SMADs	[2]
MYOSINIIA_pS1943	Up	TGF-β inhibits a decrease in phosphorylation at this site and permits retention.	[3,4]
ANNEXIN1	Up	Is known to potentiate TGF-β mediated activation of SMADs	[5]
TFRC	Up	The Transferrin receptor is known to be required for TGF-β activation	[6]
CCNB1	Up	TGF-β is known to reduce CCNB1 levels and inhibit cell cycle progression. This could be a compensatory event	[7]
CLAUDIN7	Down	TGF-β activation is known to reduce Claudin 7 expression.	[8]
E-CADHERIN	Down	TGF-β reduces E-Cadherin levels in a PI3 kinase and PTEN dependent manner.	[9]
BCL2	Down	TGF-β activation is known to reduce BCL2 levels	[10]
AR	Down	The Androgen Receptor is known to directly antagonise TGF-β signalling through colocalization with SMAD	[11]
PDCD4	Down	PD4-downregulation is known to abrogate TGF-β mediated apoptosis	[12]

Supplementary Table 6

Mutation/alteration	Association with TGF- β	Reference
TP53	needed for TGF- β cytostasis	[13]
SMAD4	Key TGF- β transcription factor	
BRAF	confers dependence on TGF-β	[14]
ACVR1B	Potentiates SMAD activation	[15]
NF1/NF2	Prior associations recorded	[16]
MYC (Amplified)	In renal fibrosis, activates latent TGF-β through integrin signalling. Also, MYC is inhibited by TGF-β activation as part of the cytostatic response, and amplification may therefore permit ability to withstand active TGF-β in the microenvironment.	[17], [18]

Supplemental References

1 Hocevar, B. A., Brown, T. L. & Howe, P. H. TGF-beta induces fibronectin synthesis through a c-Jun N-terminal kinase-dependent, Smad4-independent pathway. *The EMBO journal* **18**, 1345-1356, doi:10.1093/emboj/18.5.1345 (1999).

2 Dong, C., Zhu, S., Wang, T., Yoon, W. & Goldschmidt-Clermont, P. J. Upregulation of PAI-1 is mediated through TGF-beta/Smad pathway in transplant arteriopathy. *The Journal of heart and lung transplantation : the official publication of the International Society for Heart Transplantation* **21**, 999-1008 (2002).

3 Beach, J. R. *et al.* Myosin II isoform switching mediates invasiveness after TGF-beta-induced epithelial-mesenchymal transition. *Proceedings of the National Academy of Sciences of the United States of America* **108**, 17991-17996, doi:10.1073/pnas.1106499108 (2011).

4 Shin, J.-W., Buxboim, A. & Discher, D. E. Myosin-II Is a Major Modulator of Human Hematopoietic Stem Cell Proliferation and Differentiation. *Blood* **118**, 2343 (2011).

5 de Graauw, M. *et al.* Annexin A1 regulates TGF-beta signaling and promotes metastasis formation of basal-like breast cancer cells. *Proceedings of the National Academy of Sciences of the United States of America* **107**, 6340-6345, doi:10.1073/pnas.0913360107 (2010).

6 Lei, R. *et al.* Transferrin receptor facilitates TGF-β and BMP signaling activation to control craniofacial morphogenesis. *Cell Death & Disease* **7**, e2282, doi:10.1038/cddis.2016.170 (2016).

7 Hu, X. *et al.* TGFbeta regulates the expression and activities of G2 checkpoint kinases in human myeloid leukemia cells. *Cytokine* **37**, 155-162, doi:10.1016/j.cyto.2007.03.009 (2007).

8 Nguyen, N. *et al.* TGF-beta1 alters esophageal epithelial barrier function by attenuation of claudin-7 in eosinophilic esophagitis. *Mucosal immunology*, doi:10.1038/mi.2017.72 (2017).

9 Vogelmann, R. *et al.* TGFbeta-induced downregulation of E-cadherin-based cell-cell adhesion depends on PI3-kinase and PTEN. *Journal of cell science* **118**, 4901-4912, doi:10.1242/jcs.02594 (2005).

10 Duenker, N. *et al.* Balance of pro-apoptotic transforming growth factor-beta and anti-apoptotic insulin effects in the control of cell death in the postnatal mouse retina. *The European journal of neuroscience* **22**, 28-38, doi:10.1111/j.1460-9568.2005.04183.x (2005).

11 Chipuk, J. E. *et al.* The androgen receptor represses transforming growth factor-beta signaling through interaction with Smad3. *The Journal of biological chemistry* **277**, 1240-1248, doi:10.1074/jbc.M108855200 (2002).

12 Ozaki, I., Hamajima, H., Matsuhashi, S. & Mizuta, T. Regulation of TGF-β1-Induced Pro-Apoptotic Signaling by Growth Factor Receptors and Extracellular Matrix Receptor Integrins in the Liver. *Frontiers in Physiology* **2**, 78, doi:10.3389/fphys.2011.00078 (2011).

13 Cordenonsi, M. *et al.* Links between tumor suppressors: p53 is required for TGF-beta gene responses by cooperating with Smads. *Cell* **113**, 301-314 (2003).

14 Spender, L. C. *et al.* Mutational activation of BRAF confers sensitivity to transforming growth factor beta inhibitors in human cancer cells. *Oncotarget* **7**, 81995-82012, doi:10.18632/oncotarget.13226 (2016).

15 Staudacher, J. J. *et al.* Activin signaling is an essential component of the TGF-beta induced pro-metastatic phenotype in colorectal cancer. **7**, 5569, doi:10.1038/s41598-017-05907-8 (2017).

16 Luciakova, K., Kollarovic, G., Kretova, M., Sabova, L. & Nelson, B. D. TGF-beta signals the formation of a unique NF1/Smad4-dependent transcription repressor-complex in human

diploid fibroblasts. *Biochemical and biophysical research communications* **411**, 648-653, doi:10.1016/j.bbrc.2011.07.017 (2011).

17 Shen, Y. *et al.* c-Myc promotes renal fibrosis by inducing integrin alphav-mediated transforming growth factor-beta signaling. *Kidney international* **92**, 888-899, doi:10.1016/j.kint.2017.03.006 (2017).

18 Frederick, J. P., Liberati, N. T., Waddell, D. S., Shi, Y. & Wang, X. F. Transforming growth factor beta-mediated transcriptional repression of c-myc is dependent on direct binding of Smad3 to a novel repressive Smad binding element. *Molecular and cellular biology* **24**, 2546-2559 (2004).
