# Supplementary material for: TGF-β-associated extracellular matrix genes link cancer-associated fibroblasts to immune evasion and immunotherapy failure
Source: Nat Commun. 2018 Nov 8;9:4692. doi: 10.1038/s41467-018-06654-8 (PMC6224529; doi:10.1038/s41467-018-06654-8)
Supplement: Supplementary file 2 — Description of Additional Supplementary Files [file 41467_2018_6654_MOESM2_ESM.pdf]

Description of Additional Supplementary Files

File Name: Supplementary Data 1
Description: Table of genes differentially expressed based on limma-trend analyses comparing top and bottom quartile samples by C-ECM scores controlling for tumour type and C-ECM down score.

File Name: Supplementary Data 2
Description: Table showing results from Canonical Pathway analysis using Ingenuity Pathway Analysis for genes described in Supplementary Data 1.

File Name: Supplementary Data 3
Description: Table showing results from Upstream Regulatory analysis using Ingenuity Pathway Analysis for genes described in Supplementary Data 1.
